# Recurrent internal hip rotation gait in cerebral palsy: Case reports of two patients

**DOI:** 10.12688/hrbopenres.12893.2

**Published:** 2019-01-29

**Authors:** Rory O'Sullivan, Damien Kiernan

**Affiliations:** 1 Gait Analysis Laboratory, Central Remedial Clinic, Dublin, Dublin, Dublin 3, Ireland

**Keywords:** Cerebral palsy, internal rotation gait, internal hip rotation

## Abstract

Internal hip rotation in cerebral palsy (CP) is typically treated with a femoral derotation osteotomy. This has been shown to be largely a successful procedure but recurrence rates up to 41% have been reported. Reported risk factors include younger age, reduced hip joint impulse and ankle plantar-flexion.

We report on two patients with bilateral CP demonstrating recurrent unilateral internal hip rotation despite surgical intervention(s).  Both demonstrate a number of the reported risk factors for recurrence.  In addition, this case report specifically compared gait kinematic patterns pre and post recurrence. On comparing both patient’s hip rotation and ankle dorsi/plantarflexion kinematics they are seen to be almost identical both pre-operatively and post-operatively. Both patients appear to revert to approximately 30 ^o^ of internal hip rotation which has been shown to maximise hip abductor function. Therefore, this case report suggests that surgical derotation in isolation is unlikely to be successful in this group and we suggest that this hip and ankle pattern may help predict recurrence in unilateral internal hip rotation.

## Introduction

Cerebral Palsy (CP) is the most common cause of motor deficiency in young children occurring in 2.1 per 1000 live births ( Oskoui *et al*., 2013). Internal hip rotation gait (IHRG) is common with a reported prevalence of 31.6% in bilateral CP and is a unilateral issue in the majority (78.4%) of cases ( O'Sullivan *et al*., 2006).

IHRG in CP has been attributed to a variety of impairments associated with CP, including hip flexor, hamstring, adductor or gluteus medius tightness; femoral anteversion and hip abductor lever arm dysfunction ( Arnold *et al*., 1997; Arnold *et al*., 2000; Arnold & Delp, 2001; Delp *et al*., 1999; O'Sullivan *et al*., 2006). While the correlation between static measure of femoral anterversion and hip rotation during gait is low ( Braatz *et al*., 2013; Lee *et al*., 2013), femoral derotation osteotomy (FDRO) remains the ‘gold-standard’ treatment for IHRG ( Niklasch *et al*., 2015a; Schwartz *et al*., 2014). This is largely a successful intervention and a recent systematic review and meta-analysis has confirmed the positive effects of this surgery on the hip and pelvis during gait ( Carty *et al*., 2014) with long-term benefits reported up to 9 years post-surgery ( Dreher *et al*., 2012).

However, recurrence rates of 15% to 41% have been reported ( Church *et al*., 2017; Niklasch *et al*., 2015a) and in clinical practice these patients present a significant challenge. Recurrence of IHRG following surgery can be frustrating, presenting a dilemma for both therapist and surgeon regarding how best to preserve the effect of initial surgery or whether to consider repeat FDRO following recurrence. Being able to identify those patients likely to revert to internal hip rotation following FDRO would be of significant benefit to facilitate more informed surgical planning. If the decision is made to proceed with the FDRO the realistic potential for recurrence should be discussed with the family to manage expectations and potentially plan appropriate post-operative strategies to try and best preserve the effect of derotation.

Little is known about risk factors for recurrent FDRO and to our knowledge only two studies have reported on this. Church *et al.* ( Church *et al*., 2017) found that those more likely to recur had slower gait velocity and higher levels of spasticity. However, this is not particularly specific as a number of factors can influence gait speed. Niklasch *et al*. (2015b) reported more specific risk factors for recurrence including younger age (<10 years old), reduced hip joint impulse and increased ankle plantar-flexion and internal foot progression pre-operatively.

The purpose of this clinical case report is to highlight similarities in a recurring internal hip rotation kinematic pattern in two cerebral palsy patients despite surgical intervention(s).

## Description of the two cases

We analysed two patients with bilateral spastic cerebral palsy presenting with recurrent unilateral IHRG. Both patients were GMFCS level II meaning they could ambulate independently without assistive devices. The parents of both patients were seeking advice on possible repeat FDRO after IHRG recurred following previous intervention(s). The primary goal of any further surgical intervention was to improve the internal foot progression angle and the cosmetic appearance of gait. Both patients had an initial gait analysis prior to any surgical intervention at age five and nine years respectively. The current, most recent analysis was carried out at ages 17 and 15 years respectively. Intervening analyses were carried out following any surgical intervention demonstrating initial, short-term improvement in hip rotation but these analyses are not included in this case report.

## Initial pre-operative analysis

### Gait analysis

**Figure 1.**
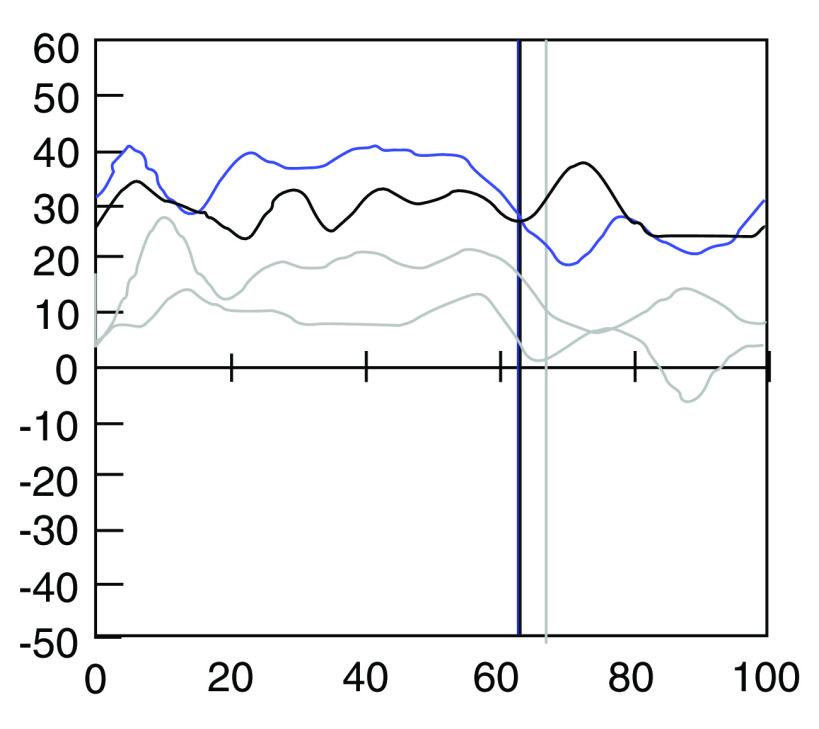
Hip rotation graphs at time of initial, pre-operative gait analysis comparing Patient A right leg-blue; Patient B left leg-black; Contralateral limbs in grey. X-axis shows percent gait cycle; Y-axis shows hip rotation (internal rotation positive; external rotation negative).

Three-dimensional kinematic and kinetic data were captured using a 4 camera Codamotion cx1 active marker system (Charnwood Dynamics, Leicestershire, UK). Kinematic data were sampled at a rate of 200 Hz while force data were captured using two Kistler force plates at a sampling rate of 400Hz. Infrared markers were placed on each participant’s lower limbs as per a modified Helen Hayes protocol ( Kiernan *et al*., 2016). Patient A demonstrated excessive internal rotation of the right hip while the left hip was more internal in Patient B. The hip kinematic graph comparing Patient A and Patient B at initial analysis demonstrates a similar degree and pattern of excessive internal hip rotation ( Figure 1). Due to young age and reduced step-lengths kinetics were not collected at the initial analysis.

### Clinical examination

Hip internal and external rotation range of movement was measured in prone lying using a gravity-reference goniometer (Myrin). Femoral anteversion was estimated in the same position using the trochanteric prominence angle test (TPAT) ( Davids *et al*., 2002).

Hip abductor strength was assessed in side lying with the knee extended and the thigh in a neutral position in terms of flexion/extension. The limb was brought into abduction and the patient asked to hold the limb in this position while progressive manual resistance was applied. Strength was scored out of a maximum of five using the modified Oxford grading ( Dan *et al*., 2014; Paternostro-Sluga *et al*., 2008). Clinical examination data at first and last assessments are summarised in Table 1.

**Table 1.** Clinical examination values at first and last assessments.

	Patient A		Patient B	
	Initial Assessment	Final Assessment	Initial Assessment	Final Assessment
Internal/External Hip Rotation (°)	80/10	75/0	95/0	80/10
Femoral Anterversion (°)	30	25	60	48
Hip Abductor Strength	3/5	3/5	4-/5	4-/5

At initial assessment, both patients demonstrated increased internal hip rotation range versus external hip rotation range and decreased strength in the hip abductors. Patient B had significantly increased femoral anteversion value at initial assessment compared to Patient A. Despite this, the dynamic hip internal rotation during gait was very similar ( Figure 1) consistent with the previously reported findings that the correlation between static measure of femoral anterversion and hip rotation during gait is low ( Braatz *et al*., 2013; Lee *et al*., 2013).

## Surgical intervention

**Figure 2.**
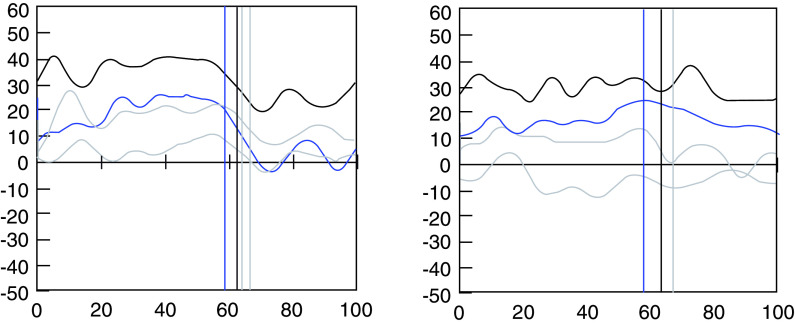
Hip rotation graphs comparing pre and post-operative analysis for Patient A (left; femoral derotation osteotomy and soft-tissue releases) and Patient B (right; femoral; derotation osteotomy). Post-operative analysis in blue; pre-operative data in black; contra-lateral pre and post-operative data in grey. X-axis of each graph shows percent gait cycle; Y-axis shows hip rotation (internal rotation positive; external rotation negative).

Patient A had a FDRO age 7 in combination with other orthopaedic procedures. The FDRO was repeated age 11 and at age 14 a surgical release of the anterior fibres of the gluteus medius was undertaken to attempt to correct the recurrent internal hip rotation. Patient B had one previous FDRO at age 11 with no additional soft-tissue releases. The current patient characteristics and past surgical histories are summarised in Table 2. In each case, post-operative gait analysis one year following intervention documented some short-term correction of IHRG but this pattern recurred in both patients ( Figure 2).

**Table 2.** Current patient characteristics and relevant surgical and gait data.

Patient	Sex	Age at last analysis	Internally rotated Limb	Surgical History	Mean Hip Rotation in Stance (°)	Mean Hip Rotation in Swing (°)	Mean Ankle Plantar-flexion in Stance (°)	Trunk Lean	Reduced Hip Joint Impulse
A	F	16	Right	Age 7: Right FDRO, Right transfer of rectus femoris to gracillis, right medial hamstring lengthening, bilateral adductor release. FDRO Age 11: Right FDRO Age 14: Release of anterior fibres of gluteus medius	33.37	22.51	0.63	Yes	Yes
B	M	15	Left	Age 11: Left FDRO	31.26	22.54	1.27	Yes	Yes

## Current post-operative analysis

### Gait analysis

**Figure 3.**
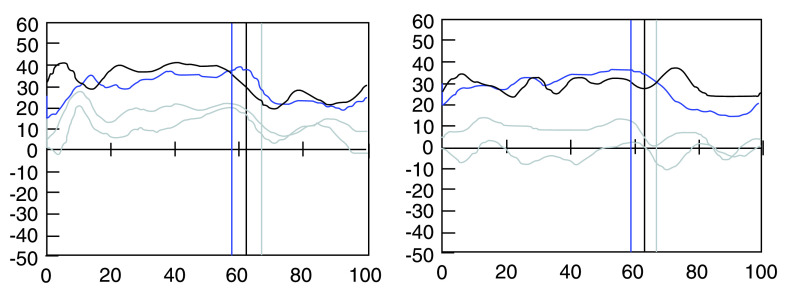
Hip rotation graphs comparing most recent gait analysis to pre-operative analysis for Patient A (left) and Patient B (right). Current analysis in blue; pre-operative data in black; contra-lateral pre and post-operative data in grey. X-axis of each graph shows percent gait cycle; Y-axis shows hip rotation (internal rotation positive; external rotation negative).

On review, current gait kinematics were compared to pre-operative gait patterns for both patients. In the case of Patient A there were 12 years between pre-operative analysis and current analysis and an interval of 5 years for Patient B. We found that the current post-operative degree and pattern of internal hip rotation were almost identical to their pre-operative data ( Figure 3).

On current gait analysis, both displayed some of the recently reported risk factors for recurrence of IHRG, namely reduced hip joint impulse and dynamic ankle plantar-flexion in the absence of ankle contracture. In addition, on video analysis, both had a significant trunk lean to the internally rotated side during stance indicative of probable hip abductor lever arm dysfunction. 

**Figure 4.**
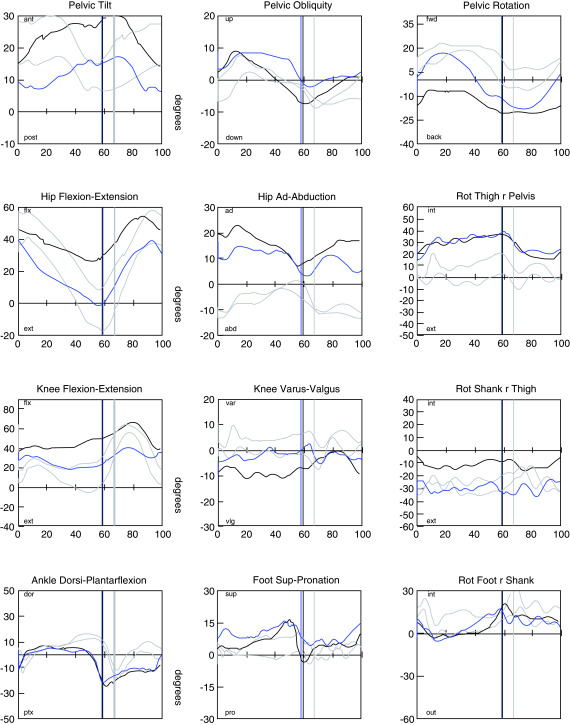
Current gait kinematic graphs for Patient A right leg-blue; Patient B left leg-black; Contralateral limbs in grey. X-axis of each graph shows percent gait cycle; Y-axis shows angular displacement. Hip rotation (internal rotation positive; external rotation negative) and ankle dorsi/plantar flexion (dorsiflexion positive; plantarflexion negative) graphs circled.

On over-laying both patients current gait kinematics we found that both had almost identical degrees and patterns of internal hip rotation and ankle plantar-flexion during gait ( Figure 4 and Table 1). This was despite otherwise different kinematics at the pelvis, hip and knee, different patient characteristics and different histories of previous surgical intervention aimed at correcting IHRG.

The hip rotation pattern in both cases demonstrates progressive internal rotation occurring through stance phase following initial contact but a significant reduction in this internal hip rotation during swing phase. Consistent with the trunk lean and reduced hip joint impulse, this kinematic pattern further suggests that the internal hip rotation is to compensate for hip abductor dysfunction as it occurs primarily when the stance phase limb is loaded but corrects when un-loaded during swing phase. 

### Clinical examination

Table 1 summarises the clinical examination findings at initial and final analysis. Despite surgical intervention, both patients continue to demonstrate significantly increased hip internal rotation versus external rotation. Femoral anteversion values have decreased compared to pre-operative values; however it must be highlighted that the reliability of the TPAT may be affected by the surgical intervention and subsequent alterations to the bony anatomy. Of note, neither patient demonstrated any change or improvement in hip abductor strength.

## Discussion

Our case report agrees with the findings of Niklasch ( Niklasch *et al*., 2015b) that reduced hip joint impulse and ankle plantar-flexion during gait appear to predict recurrence of IHRG. In addition, this case report is the first report, that we are aware of, to specifically compare the joint kinematic patterns both pre and post operatively within individual patients and also between separate patients. These comparisons show that despite documented short-term improvement post-operatively after a number of surgical interventions there was a recurrence to an almost identical position of hip rotation and ankle plantar-flexion in each case. Furthermore, it appears that the hip rotation and ankle platar/dorsiflexion kinematic patterns were very similar between the two described cases.

The similarities suggest that this pattern is somehow preferential and possibly used to maximise hip abductor function. As far back as 1965, a cadaveric study found that rotation deformities of the femur represent the most efficient use of the hip abductors ( Merchant, 1965). More recently, Arnold *et al*. ( Arnold *et al*., 1997) used musculo-skeletal modelling to suggest that 30° of internal hip rotation best restores hip abductor moment arm. This is very similar to the recurrent position of 31–34° seen in stance phase in these two patients. The relationship between ankle equinus and hip rotation has been reported in the literature ( Brunner *et al*., 2008). Therefore, we suggest that this position of ankle plantar-flexion during gait assists with passively internally rotating the hip. 

This case report primarily presents the kinematic outcomes following surgery to address IHRG in two patients. While the similarities in hip rotation kinematics are notable, the conclusions that can be drawn from this case report are obviously limited. The findings suggest that more formal research studies on larger numbers specifically comparing kinematic patterns pre and post operatively is warranted to establish if this pattern is indeed predictive of recurrence of IHRG. Based on these preliminary findings we are now examining outcomes post FDRO in a larger group. Additionally, investigation into how this pattern develops pre-operatively is suggested as, if this internal rotation is to compensate for hip abductor dysfunction, it seems likely that growth and changes in body mass index may play a role.

In terms of our current clinical practice, the findings are consistent with previous work suggesting that in a cohort of those displaying IHRG an FDRO is not likely to offer a long-lasting solution, at least in isolation. It would appear that intervention should instead be targeted at improving hip abductor capacity but at present there is no consensus on either the cause of this hip abductor dysfunction or how best to address it. Surgery aimed at altering the pull of the hip abductor muscles has been proposed but only three studies have reported on this in CP ( Cobeljić *et al*., 2005; Joseph, 1998; Steel, 1980) all of which have very small patient numbers and none have reported outcome measures using gait analysis. Therefore, we feel that the evidence does not currently exist for this intervention and so FDRO still offers the best potential for surgical correction of this gait pattern. However, while acknowledging the need for more formal research, we suggest that this gait kinematic pattern is a ‘red-flag’ for potential recurrence and this realistic possibility should be discussed with the relevant families. In addition, as hip abductor dysfunction is not addressed with FDRO we suggest specific focus on these muscle groups post-operatively and that a crutch or stick on the contra-lateral side be considered long-term post operatively to reduce the demand on the hip abductors and potentially reduce the need for recurrent internal hip rotation gait. Again though, future more formal research is needed to examine if these post-operative interventions preserve correction of IHRG.

## Conclusions

Our clinical case report highlights recurrent internal hip rotation gait in two individuals with CP despite surgical intervention(s). On specifically comparing the joint kinematic graphs we have shown that in these two case reports, the position of recurrent internal hip rotation and ankle plantar-flexion are very repeatable both within each case following surgical intervention and also between the two cases. This recurrent pattern appears to be consistent with an attempt maximize hip abductor function due to the unaddressed hip abductor weakness. While the conclusions that can be drawn from this case report are limited, we now suggest that this pattern is a potential ‘red flag’ prior to surgery which should only proceed after significant discussion on potential recurrence of IHRG. As hip abductor function is not addressed with a FDRO, post-operative rehabilitation should focus on this and a stick/crutch on the contra-lateral side may reduce the demand on the hip abductors and help preserve surgical outcomes. We suggest that more formal study on the kinematic pattern in recurrent internal hip rotation is warranted and based on this preliminary case report this work is on-going in our laboratory.

## Consent

Informed, signed consent for the use of anonymised gait analysis data was obtained from parents/guardians using our standard gait laboratory consent form and local institutional approval allows the use of such data.

## Data availability

All data underlying the results are available as part of the article and no additional source data are required.
