# Supplementary material for: Recurrent internal hip rotation gait in cerebral palsy: Case reports of two patients
Source: HRB Open Res. 2019 Jan 29;1:28. Originally published 2018 Dec 23. [Version 2] doi: 10.12688/hrbopenres.12893.2 (PMC7308961; doi:10.12688/hrbopenres.12893.2)
Supplement: Peer review history

# Peer review: Reviewer response for version 2

**DOI:** 10.21956/hrbopenres.13967.r26494

**Published:** 2019-01-30

**Authors:** Damien Bennett

**Affiliations:** 1 Public Health Agency, Belfast, Ireland

The authors have comprehensibly addressed all relevant suggestions.

Are enough details provided of any physical examination and diagnostic tests, treatment given and outcomes?

Yes

Is the background of the cases’ history and progression described in sufficient detail?

Yes

Is sufficient discussion included of the importance of the findings and their relevance to future understanding of disease processes, diagnosis or treatment?

Partly

Is the conclusion balanced and justified on the basis of the findings?

Partly

Reviewer Expertise:

Bio-mechanical engineering, public health medicine

I confirm that I have read this submission and believe that I have an appropriate level of expertise to confirm that it is of an acceptable scientific standard.

# Peer review: Reviewer response for version 2

**DOI:** 10.21956/hrbopenres.13967.r26495

**Published:** 2019-01-30

**Authors:** Mauro César de Morais Filho

**Affiliations:** 1 Pediatric Orthopedic Surgeon, Gait Laboratory and Cerebral Palsy Clinic, AACD, São Paulo, Brazil

No further comments to make.

Are enough details provided of any physical examination and diagnostic tests, treatment given and outcomes?

Yes

Is the background of the cases’ history and progression described in sufficient detail?

Yes

Is sufficient discussion included of the importance of the findings and their relevance to future understanding of disease processes, diagnosis or treatment?

Yes

Is the conclusion balanced and justified on the basis of the findings?

Yes

Reviewer Expertise:

Cerebral Palsy

I confirm that I have read this submission and believe that I have an appropriate level of expertise to confirm that it is of an acceptable scientific standard.

# Peer review: Reviewer response for version 1

**DOI:** 10.21956/hrbopenres.13962.r26460

**Published:** 2019-01-17

**Authors:** Damien Bennett

**Affiliations:** 1 Public Health Agency, Belfast, Ireland

Summary

- This useful exploratory/pilot work highlights the possibility that hip abductor lever arm dysfunction may be a risk factor for recurrence of internal hip rotation following femoral de-rotation osteotomy surgery. 
- It is suggested that post-operative rehabilitation should focus on addressing hip abductor lever arm dysfunction to help preserve surgical outcomes. 
- However, further work involving larger numbers of patients is required to test these hypotheses.

 

 This a well-defined and well described study. The study highlights the risk of recurrence of internal rotation which will be very useful to patients, their families and for clinicians involved. The take home message seems to be that unaddressed hip abductor weakness is a risk factor for recurrence and the authors suggest post-operative rehabilitation targeting the hip abductors may help preserve surgical outcomes. However it needs to be clear that this is a pilot/exploratory piece of work and this should be clear from the title, abstract, limitations section and conclusions. 

 Some other points are outlined below:

1. The main risk is of bias will come from the authors presenting 2 cases – how were they chosen? Have the authors looked at previous “failures”? 
2. The limitations of such a small number of patients needs to be highlighted to ensure that generalisability is not implied from just this study. A brief limitations section in the discussion to specifically emphasise these may be useful. This would make the conclusions more balanced.
3. Rates of 15-41% are referenced from other studies. Do the authors have estimates of failure rates for their institution?
4. The impact of age and growth at different ages would presumably also be important although may be difficult to quantify – perhaps a comment on this would be useful in the discussion. 
5. Perhaps a larger study/analysis is already being considered? – If this is the case it would be useful to emphasise this in the conclusions or as future work. 
6. Strength was scored out of a maximum of five using the modified oxford grading – give reference for the modified oxford grading system.
7. Figures - Figure 3. Patient B left leg-red – this should be black.
8. From the previous work referenced and this study it seems there are number of RFs for recurrence including slower walking speed, higher levels of spasticity, younger age, reduced hip joint impulse, increased ankle plantar-flexion and internal foot progression pre-operatively and now hip abductor dysfunction. The question is then which are the most important? It’s still likely that a case by case approach will be taken, but it seems the case made is that hip abductor lever arm dysfunction is a key new factor. To help teams in this area – how can this be most easily identified? Can this be done just from the kinematic data as shown (as well as video analysis) or do hip moments need to be considered (also see below)? 
9. The authors report “significant trunk lean and on video analysis” which suggests “probable hip abductor lever arm dysfunction”, but kinetic data is not presented here. I presume this was confirmed on hip ab/adduction moments? To demonstrate this to the reader would hip moment data be available and, if possible, would a still image be possible (assuming consent for this). Or perhaps clean force plate data was not available?

 

 Some points re possible future work are outlined below:

1. The addition of an appropriate comparator group would be key to further work which could then assess both risk and protective factors for recurrence. 
2. Regarding suggestions for a more comprehensive study this would ideally involve comparison between children who have similar gait differences and/or severity before surgery (and ideally similar characteristics such as age and possibly sex also…) and (1) had a good response to FDR Sx and those that (2) did not have a good response. The numbers here may be small, but a retrospective case control type study like this may be the only way to accurately determine what the risk factors for “failure” are. 
3. Part of a possible larger study is whether post-operative rehabilitation does prevent recurrence – presumably there are a large number of children in whom recurrence does **not** occur and is post-operative rehabilitation in these children protective?

Are enough details provided of any physical examination and diagnostic tests, treatment given and outcomes?

Yes

Is the background of the cases’ history and progression described in sufficient detail?

Yes

Is sufficient discussion included of the importance of the findings and their relevance to future understanding of disease processes, diagnosis or treatment?

Partly

Is the conclusion balanced and justified on the basis of the findings?

Partly

Reviewer Expertise:

Bio-mechanical engineering, public health medicine

I confirm that I have read this submission and believe that I have an appropriate level of expertise to confirm that it is of an acceptable scientific standard, however I have significant reservations, as outlined above.

# Response

**Published:** 2019-01-21

**Authors:** Rory O'Sullivan

**Affiliations:** Central Remedial Clinic, Ireland

We thank the reviewer for the thorough and comprehensive review and note the suggestions for further research. Firstly we would like to highlight this is a case presentation of two patients who presented similarly following recurrence of internal hip rotation. This is certainly not intended as a formal study and we were careful not to use the term 'study' in our manuscript for that reason. Based on the reviewers comments we have made this distinction more obvious through-out the paper.

Based on this case series we are moving on to more formal research on this topic and the suggestions in this regard are all valid and extremely helpful.

A number of the suggestions made by the reviewer have been incorporated into the revised manuscript.

Answers to more specific points are questions are summarized below- (our response in italics)

Some other points are outlined below:

1. The main risk is of bias will come from the authors presenting 2 cases – how were they chosen? Have the authors looked at previous “failures”? *The two cases were patients we happened to present at an orthopaedic review meeting within our centre. I over-laid the graphs as presented and was struck by the similarity. So in essence they were not "chosen" but presented but we agree that to progress to more formal research we will need to look at a more complete series of "failures" to establish how common this pattern is.*
2. The limitations of such a small number of patients needs to be highlighted to ensure that generalisability is not implied from just this study. A brief limitations section in the discussion to specifically emphasise these may be useful. This would make the conclusions more balanced. *As mentioned above we have made clearer that this is a preliminary case report that we hope might stimulate more formal research and has done in our center. *
3. Rates of 15-41% are referenced from other studies. Do the authors have estimates of failure rates for their institution? *As yet, we do not have estimates for our failure rates but this is something we are now looking to document as part of more formal study.*
4. The impact of age and growth at different ages would presumably also be important although may be difficult to quantify – perhaps a comment on this would be useful in the discussion. *Definitely agree with this point. Preliminary work seems to suggest that this pattern, in some CP children, does appear to develop secondary to growth and increasing BMI which put increased demand on the hip abductors. We have added a comment to the discussion as suggested and it is something we are examining in more formal, follow-on research.*
5. Perhaps a larger study/analysis is already being considered? – If this is the case it would be useful to emphasise this in the conclusions or as future work. *Agree and have done. *
6. Strength was scored out of a maximum of five using the modified oxford grading – give reference for the modified oxford grading system. *Done*
7. Figures - Figure 3. Patient B left leg-red – this should be black. *Done*
8. From the previous work referenced and this study it seems there are number of RFs for recurrence including slower walking speed, higher levels of spasticity, younger age, reduced hip joint impulse, increased ankle plantar-flexion and internal foot progression pre-operatively and now hip abductor dysfunction. The question is then which are the most important? It’s still likely that a case by case approach will be taken, but it seems the case made is that hip abductor lever arm dysfunction is a key new factor. To help teams in this area – how can this be most easily identified? Can this be done just from the kinematic data as shown (as well as video analysis) or do hip moments need to be considered (also see below)? *I don't think hip abductor dysfunction is newly presented in this paper as Niklash et al previously linked reduced hip joint impulse to recurrance. We agree that hip moments are useful in an individual assessment on a case by case basis. What we are suggesting, based on this preliminary report of these 2 patients, is that the kinematic pattern of approximately 30-degrees internal rotation in stance might potentially be indicative of hip abductor dysfunction as this degree on hip rotation has been shown to maximise abductor moment arm. However, more formal work is certainly required. I think that further work may highlight that CP walkers internally rotate for different reasons rather than being a homogeneous group e.g. some rotate to maximise hip abductor function and some probably just as a consequence of persistence of femoral anteversion for example. The treatment of each, and success of treatment, will depend on the cause.*
9. The authors report “significant trunk lean and on video analysis” which suggests “probable hip abductor lever arm dysfunction”, but kinetic data is not presented here. I presume this was confirmed on hip ab/adduction moments? To demonstrate this to the reader would hip moment data be available and, if possible, would a still image be possible (assuming consent for this). Or perhaps clean force plate data was not available? *It is correct that on a retrospective review of data, clean force plate data was not always available particularly in the data collected at a younger age. In the most recent analysis in each case, there was 'clean' force data and in both cases there was reduced hip joint impulse. We have not specifically included numbers or graphs of this in this case report as we feel it is beyond the scope of this preliminary report and the value to presenting 2 patients kinetic data in particular is probably questionable. For this reason, we have just highlighted it (and the trunk lean) in Table 2 but intend to examine this more formally.*

# Peer review: Reviewer response for version 1

**DOI:** 10.21956/hrbopenres.13962.r26458

**Published:** 2019-01-07

**Authors:** Mauro César de Morais Filho

**Affiliations:** 1 Pediatric Orthopedic Surgeon, Gait Laboratory and Cerebral Palsy Clinic, AACD, São Paulo, Brazil

The case report is very interesting and provocative. I believe that the paper can bring some new information for the readers.

 The Introduction is very well done and it provides a very good overview about the topic addressed for the readers.

 I believe that there is a typing mistake in page 2 at Clinical Examination Section. Please, put Oxford in page 2 (section Clinical Examination) with the first "O"on capital letter.

 Also, it would be very nice to include the short term analysis after surgical procedures showing some improvement after intervention in order to characterize the recurrence. 

 Finally, the Discussion is also very good and it stimulates further research about the same subject.

Are enough details provided of any physical examination and diagnostic tests, treatment given and outcomes?

Yes

Is the background of the cases’ history and progression described in sufficient detail?

Yes

Is sufficient discussion included of the importance of the findings and their relevance to future understanding of disease processes, diagnosis or treatment?

Yes

Is the conclusion balanced and justified on the basis of the findings?

Yes

Reviewer Expertise:

Cerebral Palsy

I confirm that I have read this submission and believe that I have an appropriate level of expertise to confirm that it is of an acceptable scientific standard.

# Response

**Published:** 2019-01-21

**Authors:** Rory O'Sullivan

**Affiliations:** Central Remedial Clinic, Ireland

We thank the reviewer for the helpful review and comments. The highlighted typing error has been corrected and a new figure has been added to the re-submitted manuscript to document the temporary improvement in hip internal rotation following surgery.

The aim of this submitted case presentation was primarily to provoke thought on this important topic in ambulant cerebral palsy and potentially stimulate further research on the topic and we thank the reviewer for highlighting this in the review.
